# Exploiting Glutamine Consumption in Atherosclerotic Lesions by Positron Emission Tomography Tracer (2*S*,4*R*)-4-^18^F-Fluoroglutamine

**DOI:** 10.3389/fimmu.2022.821423

**Published:** 2022-01-25

**Authors:** Senthil Palani, Maxwell W. G. Miner, Jenni Virta, Heidi Liljenbäck, Olli Eskola, Tiit Örd, Aarthi Ravindran, Minna U. Kaikkonen, Juhani Knuuti, Xiang-Guo Li, Antti Saraste, Anne Roivainen

**Affiliations:** ^1^ Turku PET Centre, University of Turku, Turku, Finland; ^2^ Turku Center for Disease Modeling, University of Turku, Turku, Finland; ^3^ A.I. Virtanen Institute for Molecular Sciences, University of Eastern Finland, Kuopio, Finland; ^4^ Turku PET Centre, Turku University Hospital, Turku, Finland; ^5^ InFLAMES Research Flagship Center, University of Turku, Turku, Finland; ^6^ Heart Center, Turku University Hospital and University of Turku, Turku, Finland

**Keywords:** atherosclerosis, ^18^F-fluoroglutamine, PET/CT, macrophages, inflammation

## Abstract

Increased glutamine metabolism by macrophages is associated with development of atherosclerotic lesions. Positron emission tomography/computed tomography (PET/CT) with a glutamine analog (2S,4*R*)-4-^18^F-fluoroglutamine (^18^F-FGln) allows quantification of glutamine consumption *in vivo*. Here, we investigated uptake of ^18^F-FGln by atherosclerotic lesions in mice and compared the results with those obtained using the glucose analog 2-deoxy-2-^18^F-fluoro-*D*-glucose (^18^F-FDG). Uptake of ^18^F-FGln and ^18^F-FDG by healthy control mice (C57BL/6JRj) and atherosclerotic low-density lipoprotein receptor-deficient mice expressing only apolipoprotein B100 (LDLR^−/−^ApoB^100/100^) was investigated. The mice were injected intravenously with ^18^F-FGln or ^18^F-FDG for *in vivo* PET/CT imaging. After sacrifice at 70 minutes post-injection, tracer uptake was analyzed by gamma counting of excised tissues and by autoradiography of aorta cryosections, together with histological and immunohistochemical analyses. We found that myocardial uptake of ^18^F-FGln was low. PET/CT detected lesions in the aortic arch, with a target-to-background ratio (SUV_max_, aortic arch/SUV_mean_, blood) of 1.95 ± 0.42 (mean ± standard deviation). Gamma counting revealed that aortic uptake of ^18^F-FGln by LDLR^−/−^ApoB^100/100^ mice (standardized uptake value [SUV], 0.35 ± 0.06) was significantly higher than that by healthy controls (0.20 ± 0.08, *P* = 0.03). More detailed analysis by autoradiography revealed that the plaque-to-healthy vessel wall ratio of ^18^F-FGln (2.90 ± 0.42) was significantly higher than that of ^18^F-FDG (1.93 ± 0.22, *P* = 0.004). Immunohistochemical staining confirmed that ^18^F-FGln uptake in plaques co-localized with glutamine transporter SLC7A7-positive macrophages. Collectively these data show that the ^18^F-FGln PET tracer detects inflamed atherosclerotic lesions. Thus, exploiting glutamine consumption using ^18^F-FGln PET may have translational relevance for studying atherosclerotic inflammation.

## Introduction

Atherosclerosis is a chronic inflammatory disease characterized by inflammation and accumulation of macrophages. Macrophages are highly plastic cells that play an important role during inflammation and propagation of plaques, and during the resolution of atherosclerosis (1). The vulnerability of atherosclerotic plaques to rupture correlates with the number of pro-inflammatory macrophages, whereas plaque stability correlates with the number of inflammation-resolving macrophages (2). These vital roles, together with their plasticity, make macrophages attractive targets for diagnosis and for therapy aimed at preventing or halting existing atherosclerosis.

Pro-inflammatory stimuli such as lipopolysaccharides (LPS) (3, 4) or oxidized low-density lipoprotein (5, 6) increase glycolytic capacity and glucose uptake by macrophages, phenomena that may be associated with a high risk phenotype for atherosclerotic plaques (7). This high glycolytic capacity of macrophages has been utilized to identify atherosclerotic inflammation by positron emission tomography (PET) using glucose analog 2-deoxy-2-^18^F-fluoro-*D*-glucose (^18^F-FDG) (3, 8). High glucose uptake, however, is not only a key hallmark of pro-inflammatory macrophages; it is also a common attribute of anti-inflammatory macrophages (3, 9, 10) along with all other glucose-metabolizing cells. The normally high physiological glucose uptake of myocardial cells further complicates the accurate assessment and quantification of increases in ^18^F-FDG uptake due to local inflammation. Therefore, using ^18^F-FDG PET alone as a tool to detect macrophages associated with atherosclerotic inflammation has its limitations and better tools are required for assessing the heterogeneity of macrophage activation and disease characterization.

Glutamine is an abundant amino acid and nutrient source that plays a role in exercise recovery, wound healing, metabolism, and promoting the growth of cancer cells (11). Glutamine is transported to cells by many membrane-bound solute carrier-type transporters (SLCs) (12). Experimental studies show that glutamine is required for polarization of macrophages, and that differently polarized macrophages show changes in glutamine metabolism (4, 13). A recent study demonstrated that combined assessment of 2-deoxyglucose and glutamine metabolism improved the *ex vivo* identification of macrophage polarization states (10). Furthermore, accumulation of these substrates showed different patterns in atherosclerotic lesions. However, the *in vivo* significance of glutamine uptake in atherosclerosis remains to be studied. The glutamine analog (2*S*,4*R*)-4-^18^F-fluoroglutamine (^18^F-FGln) allows quantification of glutamine consumption *in vivo* by PET. Recently, we and others showed that ^18^F-FGln is taken up preferentially by glioma cells compared with healthy brain tissue, making it feasible for *in vivo* imaging of enhanced glutamine uptake by PET (14, 15).

Here, we investigated uptake of ^18^F-FGln by inflamed atherosclerotic lesions in mice and compared the results with those obtained using ^18^F-FDG. First, we evaluated uptake of intravenously (i.v) administered ^18^F-FGln or ^18^F-FDG to detect atherosclerotic lesions using PET/CT imaging. Second, we used a gamma counter to measure the radioactivity of the administered tracer in excised tissues. Finally, we used digital autoradiography and immunohistochemistry of tissue cryosections to assess tracer accumulation in the atherosclerotic aorta and its localization in macrophage-rich lesions.

## Materials and Methods

### Chemicals and Reagents

The tosylated precursor for ^18^F-FGln synthesis and the non-radioactive reference compound FGln were provided by the Organic Synthesis Core Facility at Memorial Sloan Kettering Cancer Center, New York, NY, USA. The cassettes for ^18^F-FDG synthesis were purchased from GE Healthcare (Waukesha, WI, USA).

### Radiosynthesis and *In Vivo* Stability Analysis

The chemical structures of ^18^F-FGln and ^18^F-FDG are shown in **Supplementary Figure 1**. ^18^F-FGln was prepared according to a published method (16), with some modifications to fit into the radiosynthesis device as described in our previous work (14). Quality control of the obtained ^18^F-FGln was performed using high-performance liquid chromatography (HPLC). ^18^F-FDG was prepared in-house using a fully automated cassette-based system and a FASTLab^®^ radiosynthesis device (17). The synthesis procedure was compliant with Good Manufacturing Practices. Quality control of ^18^F-FDG before release of each batch was performed using HPLC and thin-layer chromatography.

To assess the *in vivo* stability of ^18^F-FGln, blood samples were taken at the end of PET/CT imaging. Whole-blood was weighed and radioactivity measured (Triathler 3″; Hidex, Turku, Finland) before being centrifuged at 700 ×*g* at 4°C for 5 minutes to separate the plasma. All results were decay-corrected to the corresponding animals’ time of sacrifice. A plasma sample was then weighed and radioactivity measured before a subsample was precipitated with 2.4 volumes of methanol, followed by vortexing and centrifugation at 11,000 ×*g* for 10 minutes. The radioactivity of the separated supernatant and resulting precipitated protein pellet was measured. The precipitated plasma supernatants were further analyzed using established HPLC methods (18) to measure the fraction of intact ^18^F-FGln.

### Mouse Model

To induce atherosclerosis, low-density lipoprotein receptor-deficient male mice expressing only apolipoprotein B100 (LDLR^−/−^ApoB^100/100^, strain #003000 with C57BL/6J background; Jackson Laboratory, Bar Harbor, ME, USA) were fed with a high-fat diet (HFD; 0.2% total cholesterol; TD 88137; Envigo, Madison, WI, USA) starting at the age of 2 months; this diet was maintained for 3–5 months. C57BL/6JRj male mice (7 months old; Central Animal Laboratory of the University of Turku) fed a regular chow diet were used as healthy controls. In total, 12 LDLR^–/–^ApoB^100/100^ (45.4 ± 2.4 g) and 12 healthy control mice (31.7 ± 3.9 g) were studied (**Table 1**). The mice had access to food and water *ad libitum* throughout the study, which was conducted at the Central Animal Laboratory of the University of Turku. All animal experiments were approved by the National Project Authorization Board of Finland (license numbers: ESAVI/4567/2018 and ESAVI/11751/2021) and were carried out in compliance with European Union Directive 2010/63/EU.

**Table 1:** Characteristics of the investigated animals.

	LDLR^-/-^ApoB^100/100^ atherosclerotic mice	C57BL/6JRj control mice
Age, months	5−7	6
High-fat diet, months	3−5	ND
Male animals, no.	12	12
Weight, g	45.4 ± 2.4*	31.7 ± 3.9*
*In vivo* ^18^F-FGln PET/CT, no.	4	4
*In vivo* ^18^F-FDG PET/CT, no.	4	4
*Ex vivo* ^18^F-FGln gamma counting, no.	5	5
*Ex vivo* ^18^F-FGln autoradiography, no.	5	5
*Ex vivo* ^18^F-FDG autoradiography, no.	5	4
^18^F-FGln metabolite analysis, no.	4	7
Injected ^18^F-FGln (MBq/mice)	14.5 ± 1.1*	14.4 ± 0.5*
Injected ^18^F-FDG (MBq/mice)	13.7 ± 1.2*	14.2 ± 0.3*

LDLR^-/-^ApoB^100/100^, low-density lipoprotein receptor-deficient mice expressing only apolipoprotein B100; ND, not done; no., number of investigated animals. *Values are presented as the mean ± SD.

### PET/CT Imaging

Mice were fasted for 3–4 hours, anesthetized with isoflurane (4–5% induction, 1.5−2.5% maintenance), and placed on a dedicated heating pad in the PET/CT scanner (Inveon Multimodality; Siemens Medical Solutions, Knoxville, TN, USA). The mice received i.v. ^18^F-FDG (13.9 ± 0.9 MBq) or ^18^F-FGln (14.5 ± 0.8 MBq) *via* a tail vein cannula for the 60 minute dynamic PET imaging. For anatomical reference, an iodinated intravascular contrast agent (100 µL eXIATM160XL; Binitio Biomedical, Ottawa, ON, Canada) was i.v. injected immediately after PET imaging, and a 10 minute high-resolution CT was performed. PET/CT images were analyzed using Carimas 2.10 software (Turku PET Centre, Turku, Finland; www.turkupetcentre.fi/carimas/). The regions of interest (ROI) in the aortic arch, vena cava (representing blood), and myocardium were defined using contrast-enhanced CT as an anatomical reference, as previously described (18). The myocardial ROI was consistently defined at the same site for all mice tested. The results were expressed as standardized uptake values (SUVs), which were normalized to the injected radioactivity dose and animal body weight. The maximum target-to-background ratio (TBR) at 40–60 minutes post-injection was calculated as follows: SUV_max, aortic arch_/SUV_mean, blood_ according to the established method (19).

### *Ex Vivo* Biodistribution

At 70 minutes post-injection, mice were placed under deep anesthesia, and blood samples were obtained by cardiac puncture. The mice were euthanized by cervical dislocation, and tissues were dissected and weighed. Radioactivity was measured using a γ-counter (Triathler 3″; Hidex, Turku, Finland). The gamma counting was performed on the entire aorta, extending from the aortic arch to the iliac artery bifurcation. After compensating for the remaining radioactivity in the tail and cannula, the results were expressed as SUVs, which is calculated as radioactivity concentration (becquerel per gram of tissue) normalized for injected radioactivity dose and animal body weight.

### Autoradiography, Histology, and Immunostaining

Following *ex vivo* gamma counting of excised tissues, the aorta was embedded in optimal cutting temperature compound, frozen at −70°C, and cut into 20 and 8 µm cryosections. The quantitative digital autoradiography analysis of tracer distribution was done using 20 µm cryosections, as previously described (20). The sections were exposed to a Fuji Imaging Plate BAS-TR2025 (Fuji, Tokyo, Japan) for at least 4 hours and then scanned by a Fuji Analyzer BAS-5000. After scanning, sections were stored at −70°C until staining with hematoxylin–eosin (H&E). They were then scanned with a digital slide scanner (Pannoramic 250 Flash; 3DHISTECH, Ltd., Budapest, Hungary). Tina 2.1 software (Ravtest Isotopenmessgeräte, GmbH, Straubenhardt, Germany) was used to analyze the autoradiographs. Uptake of ^18^F-FDG and ^18^F-FGln was normalized to the injected radioactivity dose per unit of body mass and corrected for radioactivity decay. Data were expressed as photostimulated luminescence per square millimeter (Normalized PSL/mm^2^).

Consecutive 8 µm sections were used to investigate co-localization of ^18^F-FGln in Mac-3-positive macrophages and glutamine transporter (SLC7A7 [solute carrier family 7, member 7])-positive macrophages. Briefly, sections were incubated with an anti-mouse Mac-3 antibody (1:1,000; catalog number: 550292; BD Biosciences, Franklin Lakes, NJ, USA) and an anti-SLC7A7 antibody (1:1,000; catalog number: PA5-113527; Thermo Fisher Scientific, Waltham, MA, USA), followed by development of a color reaction using 3.3′-diaminobenzidine (Bright-DAB, BS04-110; ImmunoLogic, Duiven, the Netherlands).

Collected mouse hearts were preserved overnight at room temperature in 10% formalin, followed by dehydration in 70% ethanol. Hearts were embedded in paraffin prior to histological characterization of atherosclerotic lesions at the level of the aortic root. Sections (6 µm thick) were cut transversely at the level of the coronary ostia, and consecutive sections were stained with modified Movat’s pentachrome or with the anti-mouse Mac-3 antibody to detect macrophages (20, 21). Furthermore, to detect SLC family glutamine transporters, sections were stained with anti-SLC1A5 (1:500; NBP1-59732; Novus Biologicals, Centennial, CO, USA), anti-SCL3A2 (1:500; sc-390154; Santa Cruz Biotechnology, Dallas, TX, USA), and anti-SLC7A7 (1:500; PA5-113527; Thermo Fisher Scientific, Waltham, MA, USA)) antibodies, followed by development of a color reaction using 3.3′-diaminobenzidine.

### Statistical Analysis

Results are presented as the mean ± standard deviation (SD). Normality was examined by a Shapiro–Wilk test, and equality of variances was tested with an F test. For normally distributed datasets, a two-tailed unpaired Student’s *t* test in Microsoft Excel was used to analyze differences between the groups. *P-*values <0.05 were considered statistically significant.

## Results

### *In Vivo* Stability of ^18^F-FGln

^18^F-FGln was prepared as previously reported, with comparable yield and purity (14). Seventy minutes after ^18^F-FGln administration, all measured parameters of metabolism in both control and diseased groups of mice were very similar. On average (*n* = 11), red blood cell uptake of the radioactivity was 46.8% ± 1.5. When analyzing plasma samples, an average (*n* = 11) of 24.6% ± 4.4 of the radioactivity was bound to proteins after approximately 70 minutes post-injection. HPLC analysis of precipitated plasma supernatant indicated that the amount of intact ^18^F-FGln in plasma was 78.2% ± 4.0 (**Supplementary Figure 2**).

### ^18^F-FGln Accumulates in Inflamed Atherosclerotic Lesions in Mice

**Figure 1:**
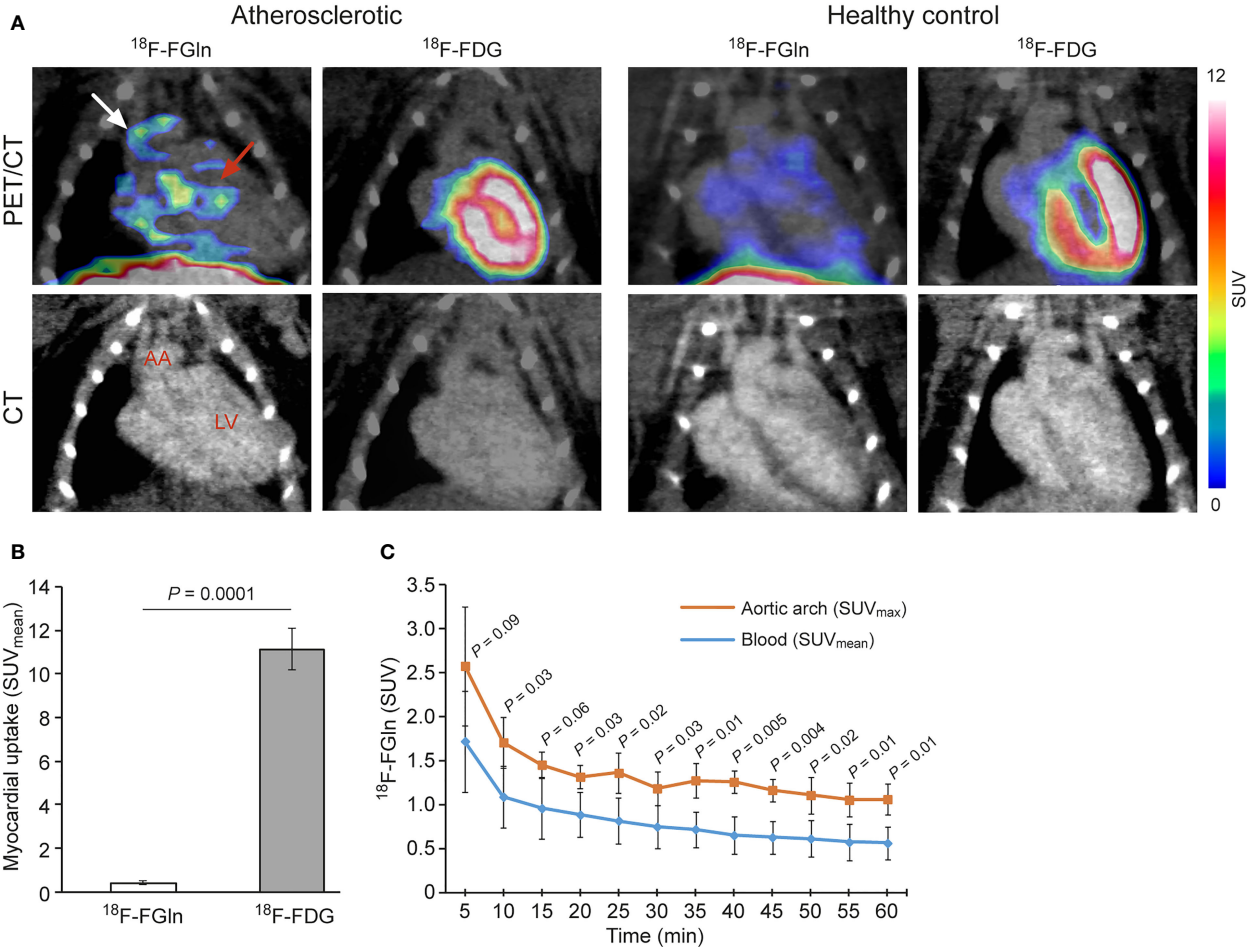
**(A)** Representative coronal PET/CT images of atherosclerotic and control mice administered with ^18^F-FGln or ^18^F-FDG. White arrow indicate the aortic arch (AA), and pink arrow indicates the myocardium. LV, left ventricle. **(B)** PET quantification of the myocardium, showing a significant difference between the tracers. **(C)** ^18^F-FGln time-activity curves in the AA and blood (vena cava) of atherosclerotic mice show a statistically significant difference (*n* = 4). Values are presented as the mean ± SD (*n* = 4). *P*-values were calculated using a two-tailed unpaired Student’s *t* test.

The *in vivo* PET/CT imaging studies of atherosclerotic mice revealed that myocardial uptake of ^18^F-FGln (SUV_mean_ 0.43 ± 0.06, *n* = 4) was significantly lower than that of ^18^F-FDG (SUV_mean_ 10.84 ± 1.10, *n* = 4, *P* < 0.0001; **Figures 1A, B**). PET/CT images showed ^18^F-FGln uptake by the aortic arch of atherosclerotic mice, whereas ^18^F-FDG was not detectable. The time-activity curve of ^18^F-FGln in the atherosclerotic aortic arch (SUV_max_) was higher than that in blood (SUV_mean_; **Figure 1C**). For comparison, all SUV_max_ and SUV_mean_ time-activity curves of ^18^F-FGln in the aortic arch and blood of atherosclerotic mice are shown in **Supplementary Figure 3**. The average TBR of ^18^F-FGln in the aortic arch of atherosclerotic mice (1.95 ± 0.42) tended to be higher than that in healthy control mice (1.44 ± 0.10, *n* = 4, *P* = 0.09). There was no difference in the TBR of ^18^F-FDG in the aortic arch of atherosclerotic mice (2.77 ± 0.71) and that of healthy control mice (2.74 ± 0.77, *n* = 4, *P* = 0.96).

*Ex vivo* gamma counting showed that uptake of ^18^F-FGln in the whole aorta of atherosclerotic mice (SUV 0.35 ± 0.06) was significantly higher than that in healthy controls (SUV 0.20 ± 0.08, *n* = 5, *P* = 0.03; **Table 2**). In both mouse strains, the highest radioactivity concentration was observed in pancreas, and the difference in this organ between LDLR^-/-^ApoB^100/100^ and C57BL/6JRj mice was statistically significant (*P* = 0.004). The lowest uptake of ^18^F-FGln was observed in brain, brown adipose tissue and white adipose tissue, respectively.

**Table 2:** *Ex vivo* biodistribution of ^18^F-FGln at 70 minutes post-injection into mice (expressed as SUV).

Tissue	LDLR^-/-^ApoB^100/100^ atherosclerotic mice (*n* = 5)	C57BL/6JRj control mice (*n* = 5)	*P*-value
Aorta	0.35 ± 0.06	0.20 ± 0.08	0.03
Brown adipose tissue	0.29 ± 0.03	0.48 ± 0.09	0.04
Bone (skull)	4.07 ± 1.56	4.62 ± 0.36	0.38
Bone + marrow (femur)	3.06 ± 1.18	3.48 ± 0.18	0.36
Blood	0.52 ± 0.10	0.51 ± 0.02	0.86
Brain	0.27 ± 0.08	0.29 ± 0.02	0.66
Heart	0.78 ± 0.18	0.96 ± 0.15	0.10
Intestine, small (empty)	2.71 ± 1.50	2.92 ± 0.49	0.94
Intestine, large (empty)	0.93 ± 0.30	1.30 ± 0.20	0.05
Kidney	2.29 ± 0.92	2.41 ± 0.31	0.74
Lungs	0.85 ± 0.22	1.07 ± 0.26	0.40
Liver	1.20 ± 0.24	1.90 ± 0.14	0.0001
Lymph nodes	0.94 ± 0.30	1.28 ± 0.11	0.06
Muscle	0.67 ± 0.28	0.91 ± 0.06	0.14
Pancreas	2.64 ± 0.62	4.77 ± 0.90	0.004
Plasma	0.60 ± 0.11	0.61 ± 0.02	0.86
Spleen	1.04 ± 0.37	1.38 ± 0.16	0.06
Stomach	1.05 ± 0.24	1.34 ± 0.20	0.04
Thymus	0.90 ± 0.38	0.94 ± 0.09	0.95
White adipose tissue	0.08 ± 0.04	0.09 ± 0.01	0.69

SUV, standardized uptake value, which is calculated as radioactivity concentration (becquerel per gram of tissue) normalized for injected radioactivity dose and animal body weight. Values are presented as the mean ± SD. P-values were calculated using a two-tailed unpaired Student’s t test.

### Macrophages in Atherosclerotic Plaques Express Glutamine Transporters

**Figure 2:**
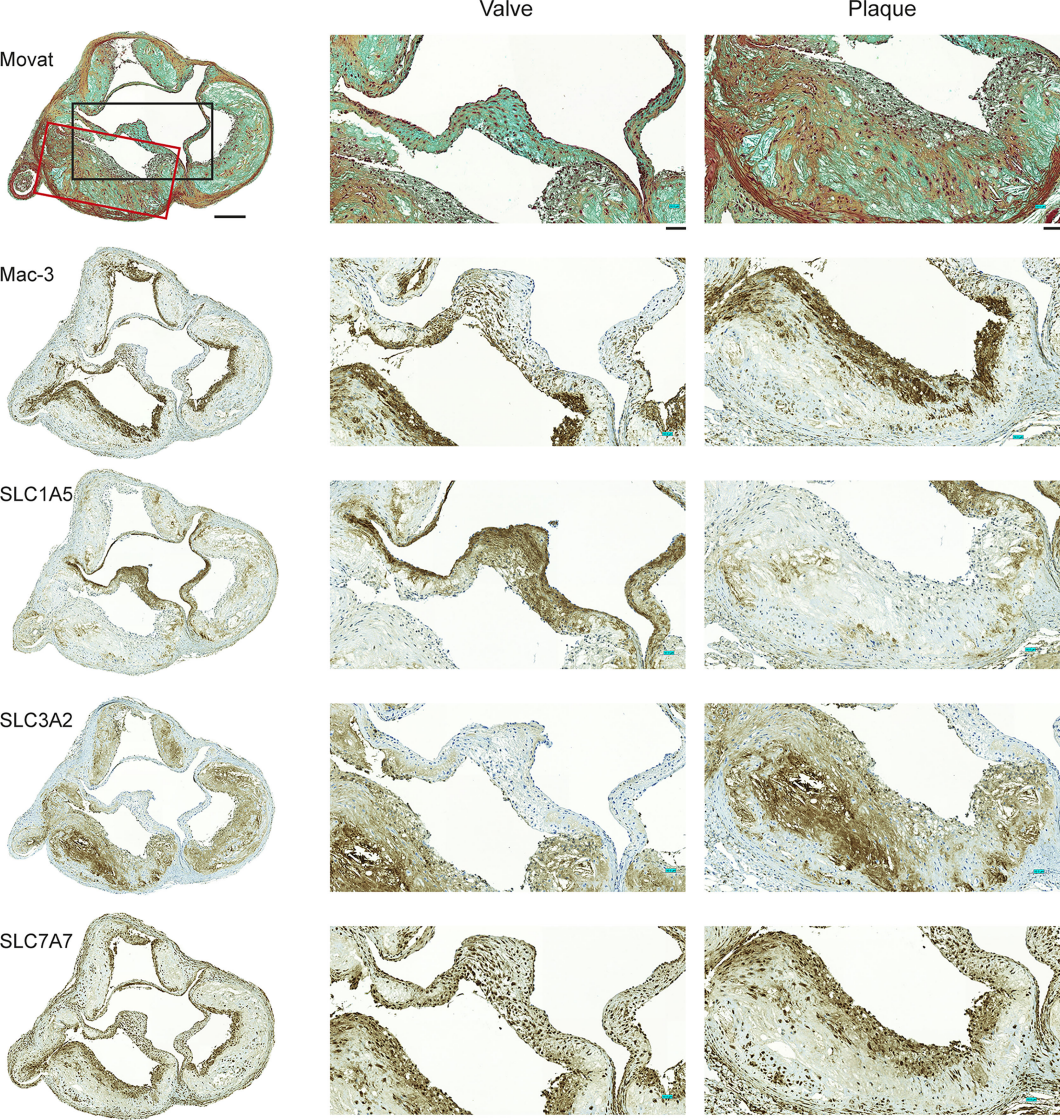
Expression of Mac-3 and glutamine transporters by mouse aortic plaque macrophages. Movat’s pentachrome staining of the aortic root demonstrates that atherosclerotic plaques were composed mostly of a fibrous cap and a necrotic region. Immunostaining of adjacent sections shows that Mac-3-positive macrophages are also positive for glutamine transporters SLC1A5, SLC3A2, and SLC7A7. Higher magnifications of the valve and plaque vessel regions are shown in the black and red rectangular boxes, respectively. Expression of SLC1A5 is prominent in the aortic valve region but not in the vessel plaque region. Expression of SLC3A2 is absent from the valve region but present in the vessel plaque region. Expression of SLC7A7 is clear in both the valve and vessel plaque regions. Scale bar = 200 µm; zoomed region scale bar = 50 µm.

**Figure 3:**
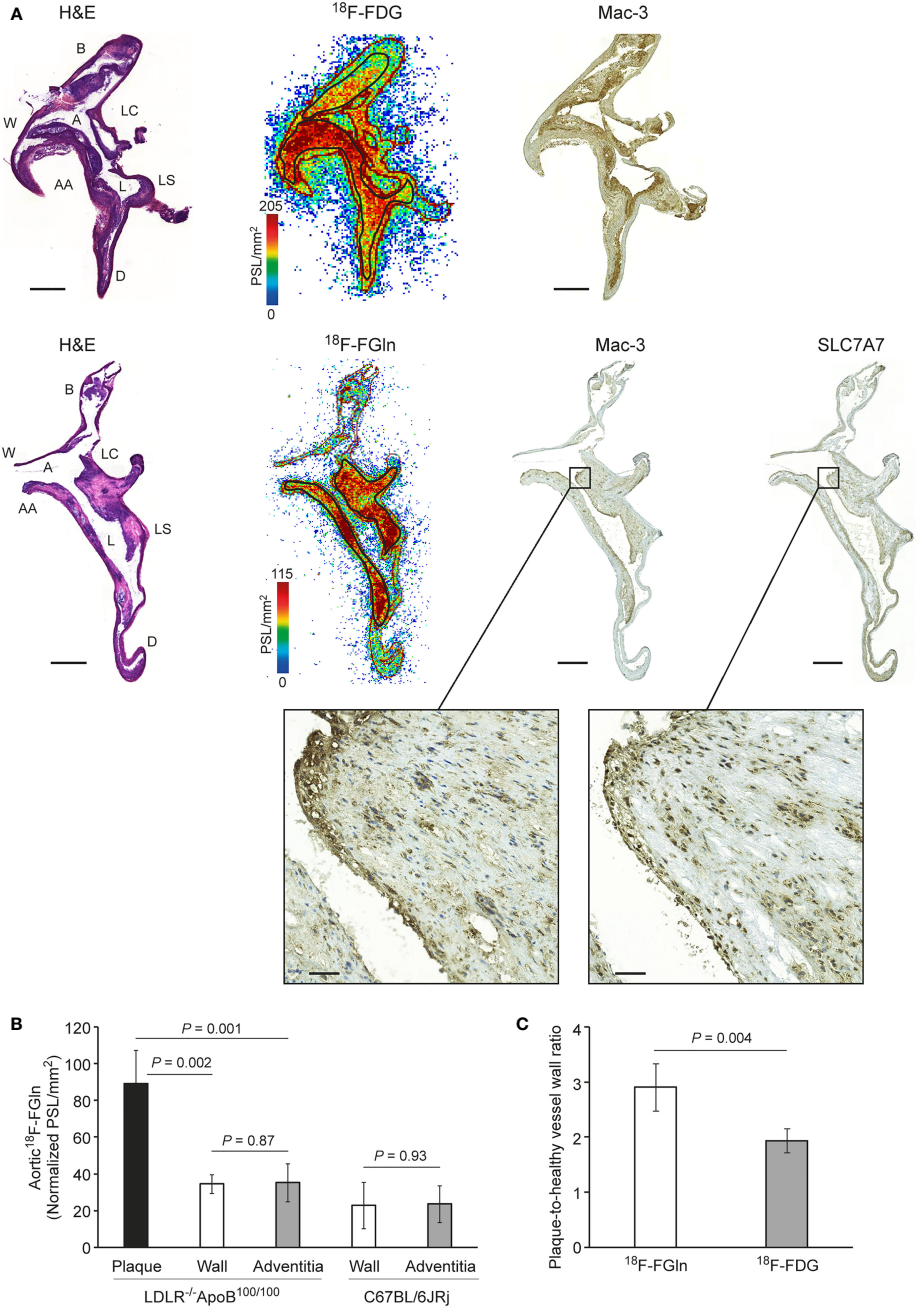
**(A)** Representative images showing hematoxylin–eosin (H&E) staining, autoradiographs, Mac-3 macrophage staining, and SLC7A7 glutamine transporter staining in consecutive aorta cryosections from atherosclerotic mice. Black rectangles denote the plaque region shown at higher magnification. Scale bar = 500 µm; zoomed region scale bar = 50 µm. A, arch; AA, ascending aorta; B, brachiocephalic artery; D, descending thoracic aorta; L, lesion; LC, left common carotid artery; LS, left subclavian artery; W, vessel wall. **(B)** Quantification of ^18^F-FGln *ex vivo* autoradiography data showing differences in tracer uptake between plaques, vessel wall, and adventitia in LDLR^-/-^ApoB^100/100^ atherosclerotic and C67BL/6JRj healthy control mice aortas. Values are expressed as the mean ± SD (*n* = 5). **(C)** Quantification of autoradiography data showing a significant difference between the tracers (*n* = 5). Values are expressed as the mean ± SD. *P*-values were calculated using a two-tailed unpaired Student’s *t* test.

Two types of samples were taken from each mouse: 1) the aorta, which extended from the aortic arch to the iliac artery bifurcation, was frozen for longitudinal cryosections, 2) paraffin-embedded aortic root was cut into cross-sections at the level of the left coronary artery ostium. According to histological and Mac-3 macrophage staining of aortic roots and aortas, the LDLR^-/-^ApoB^100/100^ mice had prominent, macrophage-rich atherosclerotic lesions, while C57Bl/6JRj mice had no signs of atherosclerosis (**Figures 2**, **3** and **Supplementary Figures 4**, **5**).

Immunostaining of aortic root sections from atherosclerotic mice showed that plaque regions were enriched with Mac-3-positive macrophages. Furthermore, SLC1A5, SLC3A2, and SLC7A7 glutamine transporters were expressed in atherosclerotic lesions, but with unique expression profiles. SLC1A5 was expressed predominantly in aortic valve leaflets, whereas SLC3A2 was expressed in atherosclerotic lesions. Expression of SLC7A7 was prominent in both the aortic valve and atherosclerotic lesions. Noticeably, SLC7A7-positive cells co-localized with Mac-3-positive macrophages (**Figure 2**). However, in control aortic roots without plaques, minimal staining with either Mac-3 or glutamine transporter antibodies was visible (**Supplementary Figure 4**).

### ^18^F-FGln Uptake Is Associated With SLC7A7-Positive Macrophage-Rich Lesions in Atherosclerotic Mice

To further elucidate the localization of ^18^F-FGln and ^18^F-FDG uptake in the aortas of atherosclerotic mice, we compared autoradiographs with histological and immunohistochemical staining. The results revealed that uptake of both ^18^F-FGln and ^18^F-FDG co-localized with Mac-3-positive macrophage-rich lesions. Notably, those macrophages were also positive for SLC7A7 (**Figure 3A**). Furthermore, detailed analysis of ^18^F-FGln uptake in atherosclerotic aortas showed that plaque regions had higher uptake of ^18^F-FGln (PSL/mm^2^ 89.05 ± 18.09, *n =* 5) than the vessel wall (PSL/mm^2^ 34.60 ± 5.23, *P* = 0.002) or adventitia (PSL/mm^2^ 35.48 ± 10.34, *P* = 0.001). There was no difference in uptake of ^18^F-FGln between the vessel wall and adventitia in either the atherosclerotic or control groups (**Figure 3B**). Furthermore, the average plaque-to-healthy vessel wall ratio of ^18^F-FGln (2.90 ± 0.42) was significantly higher than that of ^18^F-FDG (1.93 ± 0.22, *n =* 5, *P* = 0.004; **Figure 3C**). However, there was no clear tracer uptake by control aorta (**Supplementary Figure 5**).

## Discussion

Imaging of atherosclerotic lesions with ^18^F-FDG may be difficult due to physiological uptake by the myocardium and because non-inflammatory cells may consume large amounts of glucose during inflammation. Uptake of ^18^F-FGln, a glutamine analog that is used for PET imaging of cancer (22–26) correlates with upregulation of alanine-serine-cysteine transporter 2 (ASCT2), a sodium-dependent neutral amino acid transporter of glutamine (27); as such, it detects lesions more sensitively than ^18^F-FDG (23). Recently, we and others showed that uptake of ^18^F-FGln by gliomas is higher than that by healthy brain tissue (14, 15).

In addition to cancers, glutamine metabolism is altered in some inflammatory conditions. A study by Tavakoli and co-workers showed a difference in the uptake of glutamine and 2-deoxyglucose by *in vitro-*polarized macrophages (10). Macrophages polarized with IL-4 (MΦ_IL-4_) show higher uptake of glutamine than macrophages polarized with interferon-gamma and tumor necrosis factor alpha (MΦ_INF-γ + TNF-α_), or unstimulated macrophages (MΦ_0_). In the same study, an *ex vivo* experiment with ^14^C-glutamine showed uptake by macrophage-rich atherosclerotic lesions in the aortas of mice.

Here, we report for the first time that after i.v. administration, ^18^F-FGln accumulates in inflamed atherosclerotic lesions in mice, which, combined with low myocardial uptake, facilitates visualization of aortic arch lesions *in vivo* by PET/CT. The myocardial uptake of ^18^F-FGln was 25-fold lower than that of ^18^F-FDG (**Figure 1B**). Uptake of tracers by inflamed lesions was further confirmed by a more detailed analysis using *ex vivo* digital autoradiography of aorta sections, which showed a higher plaque-to-healthy vessel wall ratio for ^18^F-FGln than for ^18^F-FDG (**Figure 3C**). Immunohistochemical staining confirmed that uptake of ^18^F-FGln accumulated in plaques rich in Mac-3 and SLC7A7-positive cells. However, our results do not preclude that other cell types and glutamine transporters could be responsible for part of ^18^F-FGln uptake in atherosclerotic lesions.

To assess the *in vivo* stability of ^18^F-FGln, blood samples were collected 70 minutes after radiopharmaceutical administration and subjected to multiple assays. Very little difference was observed between control and disease populations with respect to red blood cell uptake, plasma protein binding, and the purity of the plasma fraction. Based on HPLC analysis, an average of 78.2% of plasma radioactivity detected 70 minutes post-injection was derived from intact ^18^F-FGln, indicating good *in vivo* stability.

Furthermore, immunostaining of aortic roots showed that atherosclerotic plaques were rich in macrophages (Mac-3), and that not all of the glutamine transporters are expressed uniformly in the plaque region. In line with previous studies, we observed that expression of SLC1A5 and SLC3A2 in the plaque region was not ubiquitous (10). However, we noticed that SLC7A7 expression was universal in macrophages in plaques of the aortic root, which supports the possibility that SLC7A7 is the prominent glutamine transporter in macrophage-rich plaques of the atherosclerotic aorta. Interestingly, a previous study has reported that downregulating SLC7A7 in human macrophages by using small interfering RNA triggers an inflammatory phenotype (28) suggesting SLC7A7 contribution in macrophage polarization

When we compared the plaque-to-healthy vessel wall ratio of ^18^F-FGln (2.90 ± 0.42) with that of other tracers using a similar protocol for detection of atherosclerotic lesions, we found that it was higher than that of ^18^F-FDG (1.93 ± 0.22), ^18^F-FOL (2.6 ± 0.58), ^68^Ga-FOL (2.44 ± 0.15), and ^68^Ga-NODAGA-exendin-4 (1.6 ± 0.10) (19, 29, 30).

We acknowledge that this study has some limitations. It should be noted that because the size of atherosclerotic plaques in mice is small in relation to the spatial resolution of PET scanner, spill over from adjacent tissue with low tracer uptake is likely to artificially reduce the measured uptake in small lesions. We did not perform ^18^F-FGln blocking studies *in vivo* or *in vitro*. Furthermore, we did not illustrate how glutamine consumption changes in a plaque regression model. *In vitro* blocking studies might provide insight into the specific solute carrier transporter involved in transport of glutamine during atherosclerotic inflammation. Further studies should determine whether blocking one transporter in the family might decrease uptake of glutamine, or whether blocking allows other transporters in the family to take over and compensate for any loss of glutamine uptake.

A study suggested that combined imaging of glucose and glutamine metabolism is a potential approach to better discriminate macrophage subtypes in atherosclerotic lesions with higher ratio of glutamine to 2-deoxyglucose representing the predominance of an anti-inflammatory macrophage population (10). Our autoradiography results indicate that indeed, both ^18^F-FDG and ^18^F-FGln accumulate in atherosclerotic lesions showing relatively similar distribution in macrophage-rich areas. However, we were not able to compare uptake of these tracers directly in the same atherosclerotic lesions due to limited ^18^F-FDG signal in PET images and the use of same radionuclide precluding dual tracer autoradiography. Given the feasibility of detecting atherosclerotic lesions with ^18^F-FGln, evaluation of the ratio of glutamine to 2-deoxyglucose seems a feasible approach in future studies.

The results presented herein provide preclinical evidence that ^18^F-FGln is taken up by inflamed atherosclerotic lesions in mice. Further studies using ^18^F-FGln in different atherosclerotic settings and models would strengthen data supporting the translational use of ^18^F-FGln as a tracer to image atherosclerotic inflammation.

## Data Availability Statement

The raw data supporting the conclusions of this article may be made available by the authors upon a reasonable request. Requests will be evaluated by authors. Requests to access the datasets should be directed to Anne Roivainen, anne.roivainen@utu.fi.

## Ethics Statement

The animal study was reviewed and approved by National Project Authorization Board of Finland (license numbers: ESAVI/4567/2018 and ESAVI/11751/2021).

## Author Contributions

SP acquired and analyzed the data, prepared figures, and wrote the first draft of the manuscript. MM, JV, HL, OE, TÖ, ARa, and MK acquired and analyzed the data. JK, X-GL, AS and ARo designed and supervised the work. All authors contributed to the writing and approved the submitted version of the manuscript.

## Funding

The study was financially supported by grants from the Academy of Finland (decision numbers: 314554, 314556, 335973, and 335975), the Sigrid Jusélius Foundation, the Jane and Aatos Erkko Foundation, and the Finnish Foundation for Cardiovascular Research. This research was supported by InFLAMES Flagship Programme of the Academy of Finland (decision number: 337530).

## Conflict of Interest

The authors declare that the research was conducted in the absence of any commercial or financial relationships that could be construed as a potential conflict of interest.

## Publisher’s Note

All claims expressed in this article are solely those of the authors and do not necessarily represent those of their affiliated organizations, or those of the publisher, the editors and the reviewers. Any product that may be evaluated in this article, or claim that may be made by its manufacturer, is not guaranteed or endorsed by the publisher.
