# Corrigendum: Exploiting Glutamine Consumption in Atherosclerotic Lesions by Positron Emission Tomography Tracer (2*S*,4*R*)-4-^18^F-Fluoroglutamine

**DOI:** 10.3389/fimmu.2022.902544

**Published:** 2022-04-12

**Authors:** Senthil Palani, Maxwell W. G. Miner, Jenni Virta, Heidi Liljenbäck, Olli Eskola, Tiit Örd, Aarthi Ravindran, Minna U. Kaikkonen, Juhani Knuuti, Xiang-Guo Li, Antti Saraste, Anne Roivainen

**Affiliations:** ^1^ Turku PET Centre, University of Turku, Turku, Finland; ^2^ Turku Center for Disease Modeling, University of Turku, Turku, Finland; ^3^ A.I. Virtanen Institute for Molecular Sciences, University of Eastern Finland, Kuopio, Finland; ^4^ Turku PET Centre, Turku University Hospital, Turku, Finland; ^5^ InFLAMES Research Flagship Center, University of Turku, Turku, Finland; ^6^ Heart Center, Turku University Hospital and University of Turku, Turku, Finland

**Keywords:** atherosclerosis, ^18^F-fluoroglutamine, PET/CT, macrophages, inflammation

In the original article, there was a mistake in **Figure 2** as published. The Mac-3 and SLC7A7 images on the left column were incorrectly positioned, i.e they had changed places. The corrected **Figure 2** appears below.

**Figure 2:**
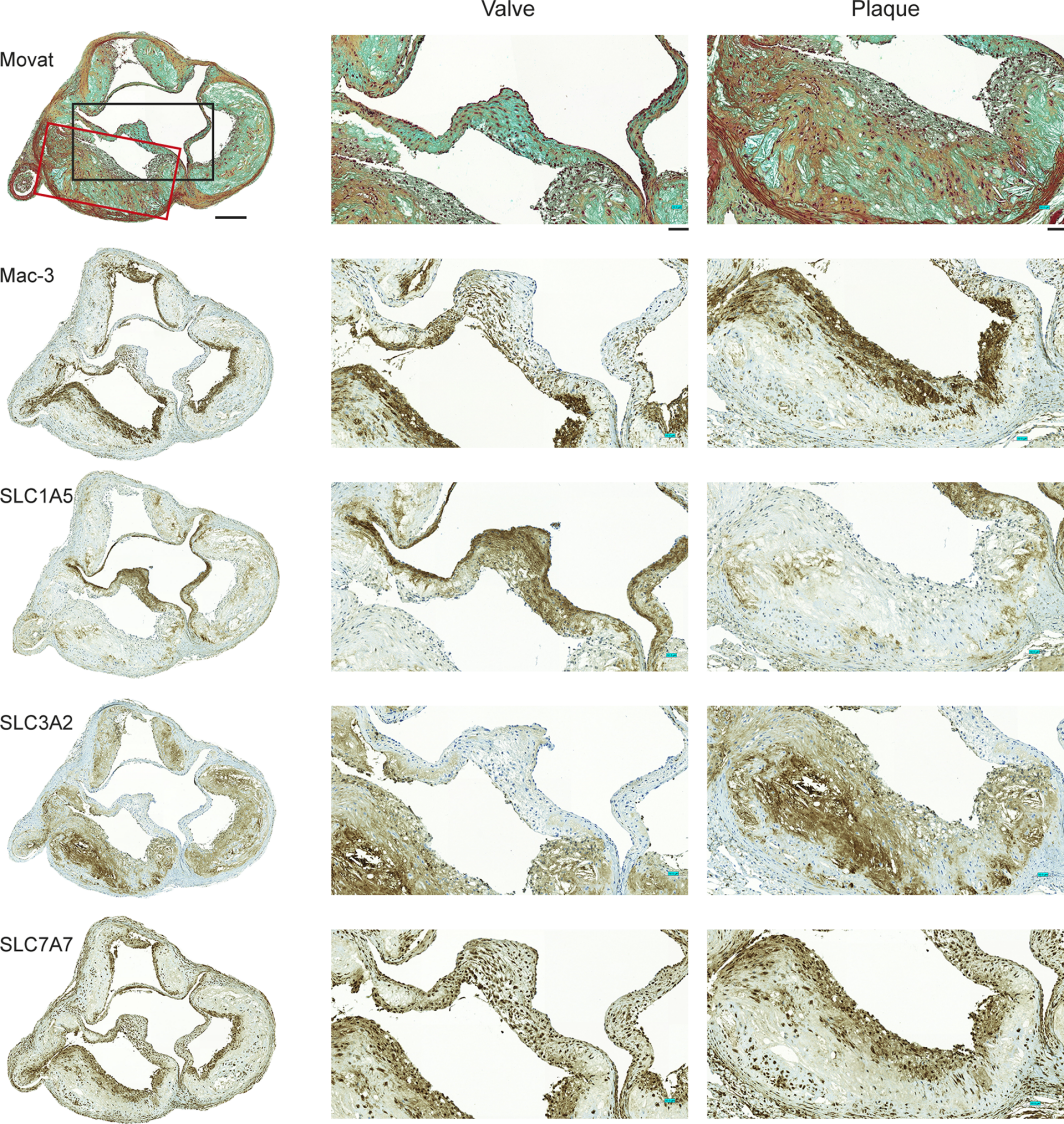
Expression of Mac-3 and glutamine transporters by mouse aortic plaque macrophages. Movat’s pentachrome staining of the aortic root demonstrates that atherosclerotic plaques were composed mostly of a fibrous cap and a necrotic region. Immunostaining of adjacent sections shows that Mac-3-positive macrophages are also positive for glutamine transporters SLC1A5, SLC3A2, and SLC7A7. Higher magnifications of the valve and plaque vessel regions are shown in the black and red rectangular boxes, respectively. Expression of SLC1A5 is prominent in the aortic valve region but not in the vessel plaque region. Expression of SLC3A2 is absent from the valve region but present in the vessel plaque region. Expression of SLC7A7 is clear in both the valve and vessel plaque regions. Scale bar = 200 μm; zoomed region scale bar = 50 μm.

The authors apologize for this error and state that this does not change the scientific conclusions of the article in any way. The original article has been updated.

## Publisher’s Note

All claims expressed in this article are solely those of the authors and do not necessarily represent those of their affiliated organizations, or those of the publisher, the editors and the reviewers. Any product that may be evaluated in this article, or claim that may be made by its manufacturer, is not guaranteed or endorsed by the publisher.

