# Supplementary material for: Exploiting Glutamine Consumption in Atherosclerotic Lesions by Positron Emission Tomography Tracer (2S,4R)-4-18F-Fluoroglutamine
Source: Front Immunol. 2022 Jan 25;13:821423. doi: 10.3389/fimmu.2022.821423 (PMC8822173; doi:10.3389/fimmu.2022.821423)
Supplement: Supplementary file 1 [file DataSheet_1.pdf]

Supplementary Material

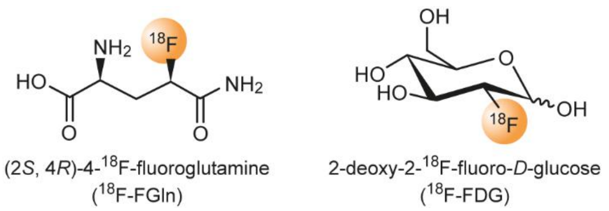

Supplementary Figure 1 Molecular structure of ^{18}F-FGln and ^{18}F-FDG.

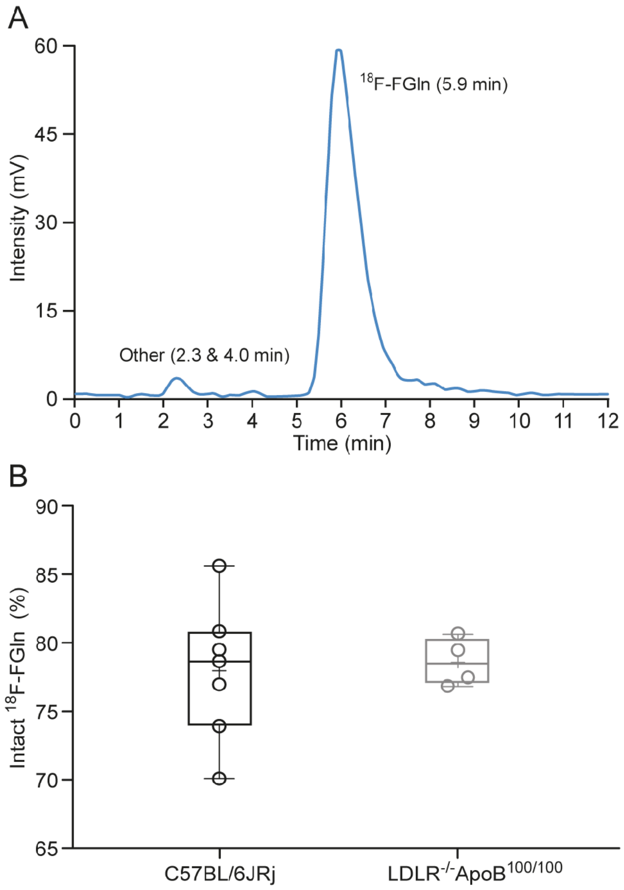

Supplementary Figure 2 (A) A sample ^{18}F-FGln HPLC flow-scintillation chromatogram showing relative precipitated plasma assay purity. **(B)** Proportion of intact ^{18}F-FGln in mouse plasma at 70 minutes post-injection, as determined by radio-HPLC analysis. C57BL/6JRj healthy control mice (*n* = 7) and LDLR$^{-/-}$ApoB$^{100/100}$ atherosclerotic mice (*n* = 4) were tested.

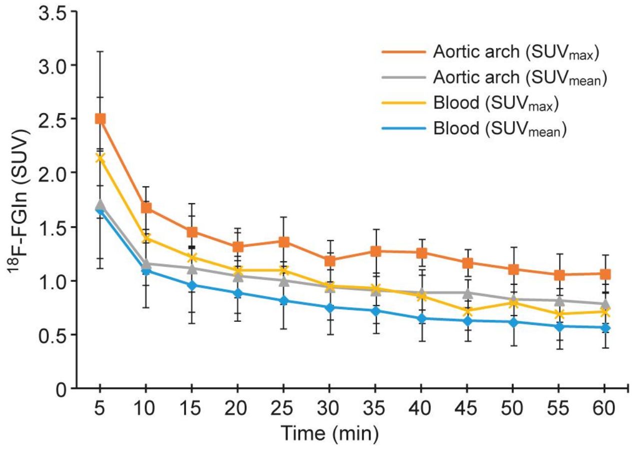

Supplementary Figure 3 SUV_{max} and SUV_{mean} time-activity curves of ^{18}F-FGln in the aortic arch and blood (vena cava) of atherosclerotic mice. Results are presented as the mean ± SD (*n* = 4).

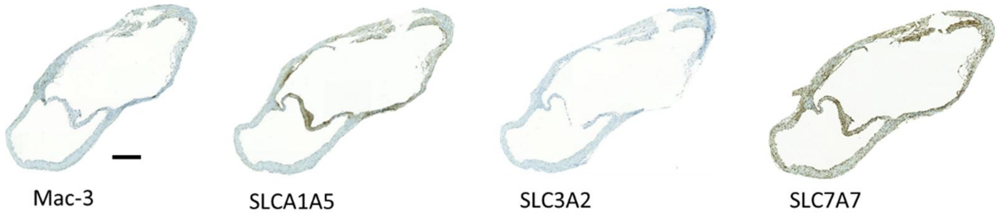

Supplementary Figure 4 Expression of Mac-3 and glutamine transporters SLC1A5, SLC3A2, and SLC7A7 in the aortic root of a C67BL/6JRj healthy control mouse. Representative images showing a clean vessel wall without plaque formation. Scale bar = 200 µm.

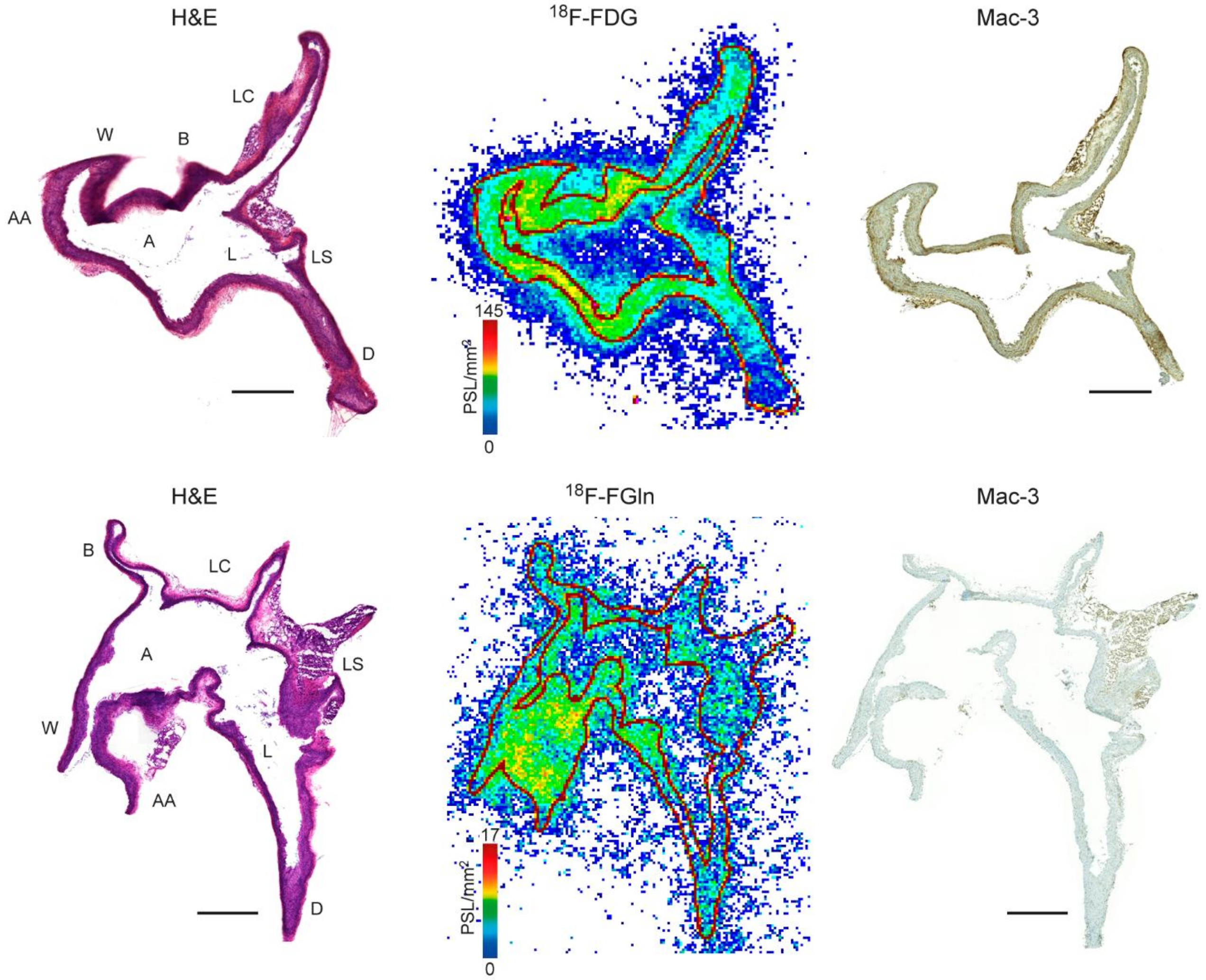

Supplementary Figure 5 Representative images of hematoxylin–eosin (H&E) staining, autoradiographs, and Mac-3 macrophage staining in consecutive aorta cryosections from a C67BL/6JRj healthy control mouse (6-month-old male fed a regular chow diet); no plaques are evident. Low tracer uptake is seen in the regions of tissue folds. Scale bar = 500 µm. A = aortic arch; AA = ascending aorta; B = brachiocephalic artery; D = descending thoracic aorta; L = lesion; LC = left common carotid artery; LS = left subclavian artery; W = vessel wall.
